# Data standards, sense and stability: Scratchpads, the ICZN and ZooBank

**DOI:** 10.3897/zookeys.150.2248

**Published:** 2011-11-28

**Authors:** Edward Baker, Ellinor Michel

**Affiliations:** 1 International Commission on Zoological Nomenclature, London, United Kingdom; 2 Department of Entomology, Natural History Museum, London, United Kingdom

**Keywords:** International Commission on Zoological Nomenclature, Scratchpads, Bulletin of Zoological Nomenclature, BioStor, BioCode, ZooBank, Global Names Architecture

## Abstract

The International Commission of Zoological Nomenclature has used the Scratchpads platform (currently being developed and maintained by ViBRANT) as the foundation for its redesigned website and as a platform for engaging with its users. The existing Scratchpad tools, with extensions to provide additional functions, have allowed for a major transformation in presentation of linked nomenclatural tools. Continued development of the new website will act as a springboard for the ICZN to participate more fully in the wider community of biodiversity informatics.

## Introduction

The International Commission on Zoological Nomenclature (ICZN: http://iczn.org) aims to provide ‘standards, sense and stability for animal names in science’ by acting as an advisor and arbiter for the zoological community. The ICZN produces the *International Code of Zoological Nomenclature* (‘the Code’) - a set of rules for the naming of animals and the resolution of nomenclatural problems. In addition it publishes the *Bulletin of Zoological Nomenclature* (BZN) containing applications (Cases) made to the ICZN, Comments on these Cases, and the rulings of the Commission (Opinions). The ICZN is also responsible for creating ZooBank (http://zoobank.org), an online repository of nomenclatural acts intended to be the official registry of zoological nomenclature, currently under consideration as a prerequisite for electronic-only publication under the Code (details of this discussion with links to associated publications can be found at http://iczn.org/content/availability-electronic-publication).

Work began in December 2009 to move the ICZN’s website to the Scratchpads (http://scratchpads.eu; Smith et al. 2011) platform. Prior to this the website was made of individual, hand-written HTML pages; with an increasing volume of content it was becoming difficult to maintain. The lack of a content management system (CMS) prevented many improvements to the site, including its visual appearance, use of metadata and development of new functionality.

Currently the ICZN finds itself at the centre of a biodiversity information crisis (e.g. Godfray 2002, 2007). The number of unrecognised taxa is estimated to be an order of magnitude more than currently described (e.g. Mora et al. 2011), legacy information is hidden behind barriers to access and shifting frames of reference, and fields such as molecular biology, not in existence at the founding of the ICZN in 1895, are presenting masses of data that is sometimes poorly contextualised and lacking a taxonomic framework. Maintaining a correct and stable nomenclature is important to all concerned with the living world, including those working in policy, public health and customs enforcement. The solution to the biodiversity information crisis will come as much from improved informatics and computer science as it will from biology - it is for this reason that the ICZN is investing considerable time in making functional and stable resources for nomenclature, and the sciences that it supports.

Another set of problems are sociological rather than technological in nature; as useful as the Code and ICZN rulings are to those who make use of zoological nomenclature, they require the acceptance of, and adoption by, the entire zoological community - from taxonomists to journal editors. A recent example of journal editors not fully understanding the requirements of the Code was the publication of *Darwinius masillae* by Franzen et al. (2009) in the (normally) electronic-only journal PLoS One. Since electronic-only publication is not allowed under the 4th Code, had the name been published as planned in e-only format, it would have been unavailable. In this instance the ICZN worked with the journal editors to create an interim solution of hard copy production of the journal for this nomenclatural act (http://iczn.org/plos).

The ICZN’s response to these challenges is to develop ZooBank as an online repository for names, and the new website (using Scratchpads) as a platform for delivering not only the BZN but for outreach to the biological (and other) communities.

The initial transfer of content to the new website was completed at the end of March 2010 and the website has continued to evolve since then. The use of Scratchpads and the underlying Drupal CMS has allowed the ICZN to create a larger, more functional online presence and begin to create, organise and disseminate its outputs in a way that is standards-compliant, scalable and allows integration with other online services (e.g. BioStor). Outreach to the zoological community has been improved by online Frequently Asked Questions, guidelines of editors of journals publishing taxonomic papers, translations of the Code into foreign languages and providing a forum for discussing the draft BioCode.

## How the ICZN uses Scratchpads

The aim of the ICZN site is to provide information about the Commission, its supporting body (the International Trust for Zoological Nomenclature), and provide access to the the Code and BZN. In this respect it differs from the majority of Scratchpad sites, which generally have a taxonomic focus. Unlike other Scratchpads, the ICZN website is not a resource built directly by a community (although Cases and Comments are written by the zoological community, only the ICZN Secretariat can add content to the site). The use of the Scratchpads platform has however allowed parts of the site to be used as a tool for community engagement (e.g. the draft BioCode).

### Customisation overview

The ICZN site builds on the functionality of a standard Scratchpads installation in a number of ways including novel use of existing tools. The most obvious of these is a new theme designed for the ICZN, as well as a number of modules that are either not enabled in a standard Scratchpad (contemplate, views_Accordion), or that have been written for the ICZN website (iczn_aker, icznblocks) - Table 1.

**Table 1.** Additional modules used by the ICZN site over a standard Scratchpads installation

**Module**	**Functionality**
contemplate	http://drupal.org/project/contemplate - Allows individual content types to be templated easily.
views_accordion	http://drupal.org/project/views_accordion - Extends the Views module functionality to provide expanadable/collapsible displays of contents. (see e.g. http://iczn.org/category/faqs/frequently-asked-questions)
iczn_aker	https://github.com/edwbaker/ICZN-Aker - Used to include (server-side) content from the private Case management Scratchpad onto iczn.org
icznblocks	https://github.com/edwbaker/ICZN-Blocks - Provides the tabbed block on the iczn.org home page. Makes use of jQuery UI to provide transitioning effects.

## Bulletin of Zoological Nomenclature

The Bulletin of Zoological Nomenclature (BZN: http://iczn.org/bzn) publishes Cases sent to the ICZN, Comments on these Cases and the rulings of the Commission (Opinions). Information relating to an individual Case is therefore spread out over several issues of the BZN. This, combined with the fact that taxonomists are generally interested in particular taxa, means that the traditional journal browsing structure of volume/issue/article is not necessarily the best way for visitors to find content. Previously BZN content was displayed as a series of Tables of Content for individual issues, and individual articles did not have their own page or associated metadata.

### BZN: Improvements

**Browsing**

Visitors are able to browse the content of the BZN by major taxonomic group (a restricted vocabulary the ICZN has used for many years) and Case number in addition to the standard volume and issue. Browsing by Case allows the entire published history of a Case to be accessible on a single page (e.g. http://iczn.org/case/3455). This is the first time this has been achieved and demonstrates the clear advantage of digital management of distributed information such as nomenclatural cases and judgements.

**Communication**

Having the BZN online in a structured form for the first time has allowed the ICZN to automatically alert users automatically to new content by e-mail or RSS feed (http://iczn.org/content/notification-cases-comments-and-opinions).

Creating an account on the site allows the user to subscribe to e-mail notifications for all BZN content, or a subset defined by the ICZN’s taxonomic groups.

**Digitisation and metadata creation**

The ICZN website has full bibliographic data for all BZN papers from Volume 63 (2006) to the present. In addition, the full text of Comments is also available (Cases and Opinions only have abstracts available).

The Biodiversity Heritage Library (BHL: http://www.biodiversitylibrary.org/bibliography/51603) has scans of Volumes 1-67, although it has no article-level metadata for these scans. The ICZN is using Rod Page’s BioStor (http://biostor.org/issn/0007-5167) tool to collect metadata for those volumes for which we have no data for at present. Once data collection is completed, an export from BioStor will be used to populate the missing volumes on the ICZN website. These articles will have a link back to the article on the BioStor site (e.g. http://biostor.org/reference/66840) where visitors will be able to view the relevant pages from BHL, or download the article in PDF format.

In the near future it is planned to release a set of simple instructions for people who would like to contribute to BZN metadata creation on BioStor; this will expediate collection of these data through crowd-sourcing.

### BZN: Technical implementation

BZN papers belong to one of four categories; (General) Articles, Cases, Comments or Opinions. All papers are entered into the standard Drupal Biblio module (http://drupal.org/project/biblio).

Several other Scratchpads are used as an online platform for journals. The European Mosquito Bulletin (http://e-m-b.org) has many articles online, however there is no article-level metadata which is an essential requirement for the ICZN, and easily achieved with the Drupal biblio module which is part of the standard Scratchpads profile. A more scalable and functionally robust system has been used for the journal Phasmid Studies (http://phasmid-study-group.org/category/PSG-Publications/1165), storing article data using the biblio module, and creating a browsable volume/issue hierarchy using a standard Drupal vocabulary.

In an extension of the method used by Phasmid Studies the browsing of the BZN by volume/issue, taxonomic group and Case number is facilitated by the use of three separate vocabularies. These vocabularies are browsed using the Scratchpads TinyTax taxonomy browser (originally intended to navigate biological taxonomies). Pages relating to terms in these taxonomies are generated dynamically using the Mado module (originally designed to display species pages) and a small number of custom views.

### BZN: Future plans

The use of web technologies can bring three key improvements to the BZN in the future:

1. easier submission of Cases

2. shorter time between a Case being submitted and an Opinion being issued

3. wider reach and community involvement

Online submission of Cases and Comments is a priority for the ICZN. The method we use to implement this functionality must integrate closely with the website, allowing papers submitted and edited online to be published to the site with a single click once they have passed the review. The Scratchpads platform already has support for the creation of papers and their electronic submission to a journal (Blagoderov et al. 2010) and it is hoped that we can adapt this process to fit our existing editorial and publication protocols.

It is hoped that the amount of time between a Case being published and an Opinion being issued will be reduced by allowing the pre-publication of Comments accepted for publication on the ICZN website. This is already being trialled for selected Comments (http://iczn.org/preprints).

Expanding the reach of the ICZN and increasing community involvement can be partially achieved by extending the automatic notification of new BZN papers by RSS or e-mail to mailing lists (e.g. Taxacom, ICZN-List). By expanding the at-present crude (although functional for a print journal) classification to order or family level for new Cases it will be possible to customise these alerts to contain only notifications about a given taxonomic group. This customisation would allow the editors of taxon-specific journals to easily publish details of new Cases, and for individual scientists to be made aware of Cases that might impact the nomenclature of ‘their’ group. Linking these feeds to social media platforms such as Facebook and Twitter will allow further dissemination of information to interested parties and help consolidate the ICZN’s current social media presence.

The online BZN will be enhanced with additional XML metadata to allow nomenclatural acts to be automatically included in ZooBank. The XML schema to be used is currently under consideration by the ZooBank developers in consultation with Pensoft and others.

## Case management

### Case management: technical implementation

The ICZN uses a separate and private Scratchpad (Aker: http://aker.iczn.org) to manage data about Cases. A custom content type holds basic information about each Case and it’s progress from submission, through publication, voting by the Commission and finally the publication of a Commission ruling (Opinion).

Customised views in Aker provide HTML content to the ICZN website. Currently this information is limited to a list of Case submissions that have yet to be published (new Cases) and Cases currently accepting Comments (open Cases). The use of the matrix editor allows batches of Cases to be edited simultaneously, which is particularly useful when it comes to advancing Cases through the system on BZN publication dates.

Aker provides information on new and open Cases in HTML format to the ICZN website. Using XHTML as the transfer format makes it easier for other people to re-use this content on their own sites either using an HTML iframe or a server-side solution.

The HTML is generated by using the ‘XML data document’ style in the Views module. The style options used to generate the HTML document are as follows:

Root element name - ul

Top-level child element name - li

Content-type - text/html

These settings result in a standard HTML unordered (bulleted) list.

A custom module, iczn_aker, is used on the ICZN website to provide a server-side solution to the display of these views.

### Case management: future plans

We are planning to develop a system for online submission that integrates with the private Scratchpad, allowing for seamless online management of Cases. Similar technology already exists in the Scratchpad’s publication module, but is likely to need extensive customisation as we need not only to allow for the creation and submission of articles, but also to see them through review and publication.

The ability to filter the XHTML output of Case information by the ICZN’s taxonomic grouping would allow external websites to include details of relevant new and open Cases dynamically. Adding the ability to search by taxon name would allow details to be displayed on dynamically created taxon pages, such as those used generated by Scratchpads and SpeciesFile.

### The Code

The International Code of Zoological Nomenclature is a set of rules and protocols for the naming of animals.

### The Code: future plans

The Code is a long and technical document that can be challenging or intimidating to first-time users. The ICZN Secretariat has written concise instructions for journal editors publishing nomenclatural acts that explains what they must do to meet the Code’s requirements. It is hoped that this will be the first of several sets of guidelines. Although these documents will stand in their own right, they must also be integrated with both the ICZN Scratchpad and the online Code.

At present, foreign-language versions of the Code are not presented via the same interface as the official code. As Drupal has in-built support for translations in the long-term there is a possibility that this could function as a platform for both the transcription and display of future translations.

There is currently an Editorial Committee charged with writing a new edition of the Code. Discussions are active on whether the revised Code could be streamlined and simplified by more dynamic, linked structure. Development of the new Code in conjunction with the Scratchpads structure could present technical improvements that make the Code a more widely accessible tool.

## Official Lists & Indexes

The Official Lists and Indexes of Names and Works in Zoology (ICZN 1987; Supplement: ICZN 2001) give details of the names and works (publications) ruled upon by the ICZN. Although when dealing with paper publications such lists and indexes provide a useful, if not essential, entry point to the BZN, with an improved and metadata-rich online presentation of the journal the need for separately maintained entry points is eliminated. The expanded metadata required to allow harvesting of BZN papers by ZooBank will provide the information required to create an automatically updated, searchable version of the print publication that can also be filtered by taxonomic group and that links directly to the Case history and associated Opinion.

## Draft BioCode

The 2011 draft BioCode aims to “provide an over-arching common framework” (http://www.bionomenclature.net/biocode2011.html) for biological nomenclature, working alongside the existing (or special) Codes. The 2011 draft BioCode was published in various journals and websites (e.g. Greuter et al. 2011). In order to make it clearer how the BioCode relates to the special Codes an article level treatment was created on the ICZN Scratchpad (http://iczn.org/biocode) showing the BioCode article alongside relevant articles from, and links to, the special Codes.

Although the draft BioCode is not an output of the ICZN it was decided that providing a forum for its discussion would benefit the zoological community and improve response. People wishing to contribute to the discussion can create a free account (http://iczn.org/user) and leave comments on individual articles. It is hoped that grouping articles from the draft BioCode and special Codes together in a thematic format will provide a useful resource not only for putting the draft BioCode in context but also for comparisons of style, language, structure and methodology between the existing Codes.

### Draft BioCode: technical implementation

BioCode articles are linked to articles form the special Codes via a standard nodereference field.

The collapsible/expandable views are created using the Views Accordion plugin for the Drupal Views module. These are included on BioCode article pages using the Contemplate module and the views_embed_view() function. This functionality is not enabled by default on Scratchpad sites as it would allow site owners to run arbitrary PHP code.

## Outreach

In addition to the technical procedures outlined above the use of a CMS has allowed more time for additional content to be made available online. The bulk of this content has focused on bridging the sociological gap between working biologists and the nomenclatural community. Examples of this work include a series of Frequently Asked Questions (http://iczn.org/faqs), some educational resources about nomenclature (http://iczn.org/education_outreach), videos of ICZN sponsored events (http://iczn.org/video) and even a PodCast (http://iczn.org/podcast).

## The future

“The Linnean Enterprise has persisted for two and a half centuries, and the ICZN Code is itself more than a century old” (Pyle and Michel 2010).

The ICZN has responsibilities that go far beyond serving the current generations of zoologists. It must also honor and preserve the “robust historical legacy” (Pyle and Michel 2010) of existing zoological nomenclature whilst also ensuring a stable platform for its long-term future. This can only happen by working with the zoological community in its entirety.

Through ZooBank the ICZN will help to create the Global Names Architecture - allowing taxonomic and other biological resources across the web to connect to each other and create a resource far greater than the sum of its parts. The ICZN website will continue to expand, not just as a destination for people to find information, but as an active platform and arena for the zoological and other nomenclatural communities to converse with the Commission and each other.

The Scratchpads platform has enabled the ICZN to make large strides towards its goals, both technical and sociological. In the future we wish to build upon this relationship, both as a user of, and contributor to, the Scratchpads platform and via ZooBank as an engaged participant of the global biodiversity informatics landscape.
